# Keratoacanthoma Pathobiology in Mouse Models

**DOI:** 10.3390/diseases2020106

**Published:** 2014-05-23

**Authors:** Katherine N. Gibson-Corley, Laura M. Rogers, Adam Goeken, Adam J. Dupuy, David K. Meyerholz

**Affiliations:** 1 Department of Pathology, University of Iowa, Iowa City, IA 52242, USA; 2 Holden Comprehensive Cancer Center, University of Iowa, Iowa City, IA 52242, USA; 3 Department of Anatomy & Cell Biology, Roy J. & Lucille A. Carver College of Medicine, University of Iowa, Iowa City, IA 52242, USA

**Keywords:** keratoacanthoma, mouse models, histopathology, regression

## Abstract

Recently we described skin tumors driven by skin-specific expression of *Zmiz1* and here we define keratoacanthoma pathobiology in this mouse model. Similar to human keratoacanthoma development, we were able to segregate murine keratoacanthomas into three developmental phases: growth, maturation, and regression. These tumors had areas with cellular atypia, high mitotic rate, and minor local invasion in the growth phase, but with development they transitioned to maturation and regression phases with evidence of resolution. The early aggressive appearance could easily be misdiagnosed as a malignant change if the natural pathobiology was not well-defined in the model. To corroborate these findings in the *Zmiz1* model, we examined squamous skin tumors from another tumor study in aging mice, and these tumors followed a similar biological progression. Lastly, we were able to evaluate the utility of the model to assess immune cell infiltration (F4/80, B220 Granzyme B, CD3 cells, arginase-1) in the regression phase; however, because inflammation was present at all phases of development, a more comprehensive approach will be needed in future investigations. Our study of keratoacanthomas in selected murine models suggests that these squamous tumors can appear histologically aggressive during early development, but with time will enter a regression phase indicating a benign biology. Importantly, studies of squamous skin tumor models should be cautious in tumor diagnosis as the early growth distinction between malignant *versus* benign based solely on histopathology may not be easily discerned without longitudinal studies to confirm the tumor pathobiology.

## 1. Introduction

Squamous cell tumors of the skin can, at times, cause challenges in diagnostic histopathology to distinguish benign *versus* malignant (e.g., squamous cell carcinoma) behavior because of overlapping morphologic features and markers, as well as inability to follow the natural course of disease in human subjects [1]. Many of these diagnostic challenges can also extend into the evaluation of cancer in animal models. However, the diagnostic relevance of a histopathological “signature” in a tumor from an animal model should be clarified through validation studies following the natural progression of these tumors [2].

Keratoacanthomas (KAs) are skin tumors classically characterized as crateriform masses filled with abundant keratin material and lined by proliferative stratified squamous epithelium [3,4]. In humans they tend to occur on areas of the skin exposed to ultraviolet light, such as the face, hands, and forearms [5]. These are first clinically recognized as small raised masses that can enlarge fairly quickly over the course of months and then most often regress with complete disappearance by two to nine months [4].

Three distinct phases of KA growth have been described both clinically and histologically in humans: growth, maturation and regression [4,6]. In the growth and maturation phases, KAs can display local invasion along with cellular pleomorphism and proliferation, which can make them challenging to differentiate from squamous cell carcinoma (SCC) [1]. Moreover, in some cases, KAs have been reported to be a low grade malignancy or transform to SCC, further confusing the distinction between malignant and benign diseases [4,7]. As a result, KAs have a history of controversy regarding their biology and clinical relevance. Similar issues can confound animal models of cancer and their diagnoses. In this study of murine KAs, we wanted to validate tumor biology and develop standardized morphologic definitions for the different phases of tumor development for future translational investigations.

## 2. Materials and Methods

### 2.1. Mice

We studied cutaneous squamous tumors from two different genetically engineered mouse models. Zmiz1^Δ1-185^;K14-Cre double transgenic mice (Zmiz1) generated KAs as previously described [8]. We also studied tumors from a *Sleeping Beauty* (SB) transposon mutagenesis screen performed in Rag-2-deficient mice (B6.129S6-*Rag2^tm1Fwa^* N12; or Rag2^−/−^, Taconic, Hudson, NY, USA) and heterozygotes (RAG2^+/−^) (both abbreviated “Rag-SB”). [9]. Squamous skin proliferations were excluded from this study if they had (1) reactive acanthosis or (2) overt squamous cell carcinoma that lacked evidence of originating from a KA-like lesion. All animal use in this study was approved by the University of Iowa Animal Care and Use Committee (IACUC). Animals were euthanized when morbidity or tumor burden reached a predefined threshold as approved by the UI-IACUC, thus the ability to follow all tumors through regression was not feasible and therefore only approximately 14% of tumors were within the regression phase at the time of the study's endpoint.

### 2.2. Clinical Tumor Distribution

Mice from the Zmiz1 (n = 33) and Rag-SB (n = 42) groups were examined for predilection of tumor development. In both studies, comprehensive necropsies and histopathological analyses were performed [8,9]. Distribution of KAs was recorded and grouped according to regional anatomic sites (Table 1).

### 2.3. Histopathology

Skin tumors from the Zimz1 and Rag-SB studies described above were excised at necropsy and immersion fixed in 10% neutral buffered formalin. Pilot studies suggested that deep margins were the principal sites to evaluate for invasive tendencies in the murine KAs. Accordingly, tumor excision with lateral (~2 mm) and deep margins (~3 mm) were targeted. Complete necropsies were performed on all animals and importantly, draining lymph nodes and lung samples were evaluated and did not show evidence of metastasis.

Following fixation (approximately 3–5 days) tissues were routinely processed, paraffin-embedded, sectioned at 4 μm and stained with hematoxylin and eosin (HE). For this study, murine tumors were histopathologically examined by two board-certified veterinary pathologists. Based on KA literature, tumors were evaluated for morphologic features that paralleled the phases of KA development in humans (Table 2).

### 2.4. Histologic grading

KAs from Zmiz1 (n = 90) and Rag-SB cohorts (n = 47) were selected for this study based on size (broad range of small to large tumors) and quality of the histologic preparation (appropriate sectioning to be able to see tumor margins, *etc*.) to give a broad repertoire for examination. Each tumor was evaluated and scored after mutual agreement of two pathologists and by following the general principles of histopathologic scoring of data [10]. Each tumor was given a clinical “KA score” related to the KA development phase as defined in Table 2. Those tumors in the “growth” phase were given a score of “1”, those in the “maturation” phase were given a score of “2” and those in the “regression” phase were given a score of “3”. The tumors were also scored based on the estimated percentage of tumor mass that was composed of keratin (“keratin score”) using a scale from “0” (0%–10% of tumor filled by keratin), “1” (11%–20% of tumor filled by keratin), and so on to “10” (91%–100% of tumor filled by keratin). Finally, KA “diameter” (surface edge to surface edge of tumor) in millimeters (mm) was measured for all of the tumors. These scores were evaluated and linear regression statistical analysis was performed using GraphPad Prism software.

### 2.5. Immunohistochemistry

As an initial validation to define infiltrating inflammatory cells we selected one tumor in the regression phase from each genotype to do immunohistochemistry (n = 3 KAs total). Inflammatory cell markers (CD3, B220, F4/80, Arginase-1, Nos2, and granzyme-B) were assessed and these were performed on the formalin-fixed paraffin-embedded samples as summarized in Table 3. All immunohistochemical staining was performed manually using peroxidase methods and Dako Envision systems (Glostrup, Denmark).

## 3. Results

### 3.1. Pathological Appearance of Murine Keratoacanthomas

**Figure 1:**
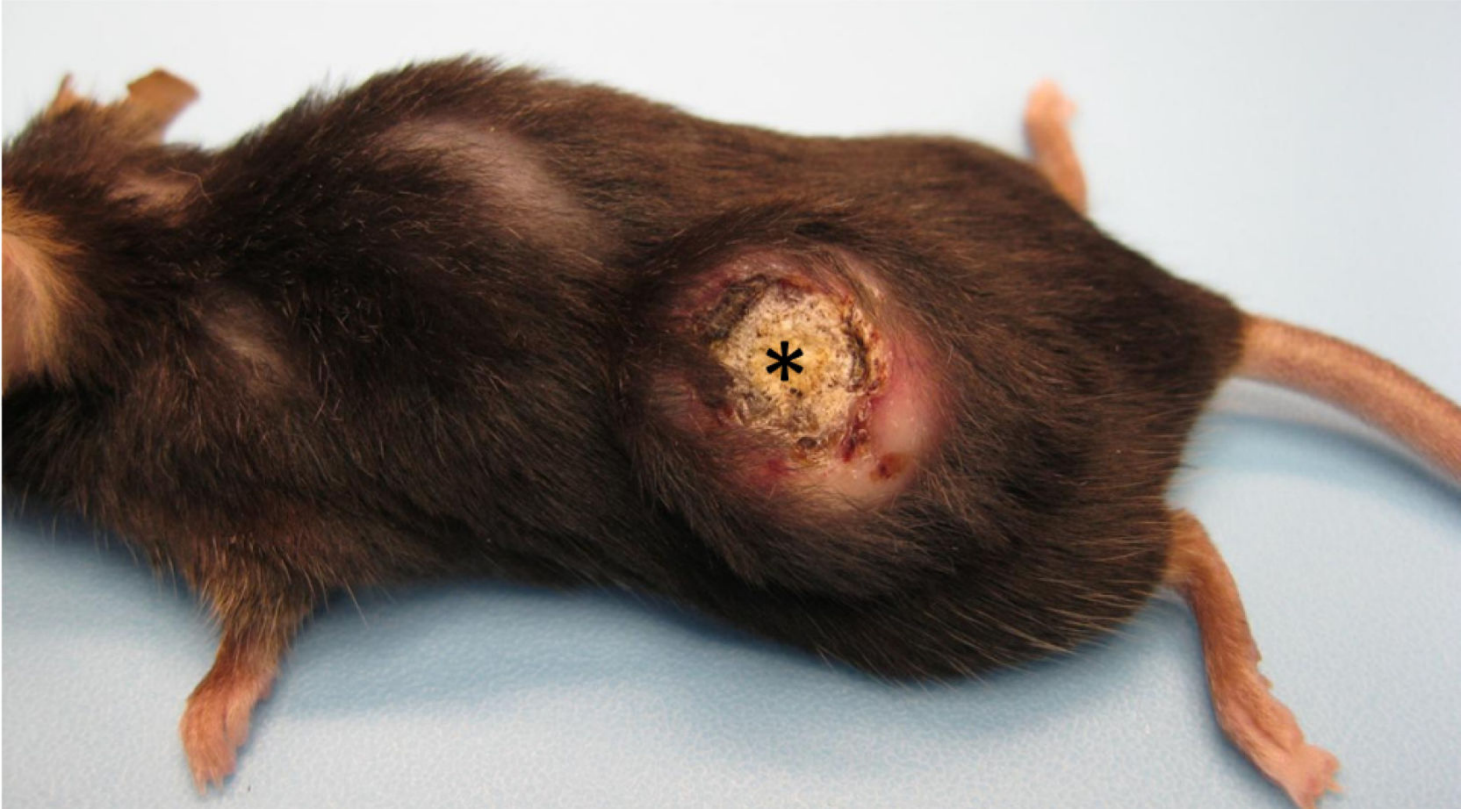
Gross appearance of a mature keratoacanthoma from a *Zimz1* mouse. The circular tumor is composed of a central core of keratin (asterisk) surrounded by raised skin margins.

**Figure 2:**
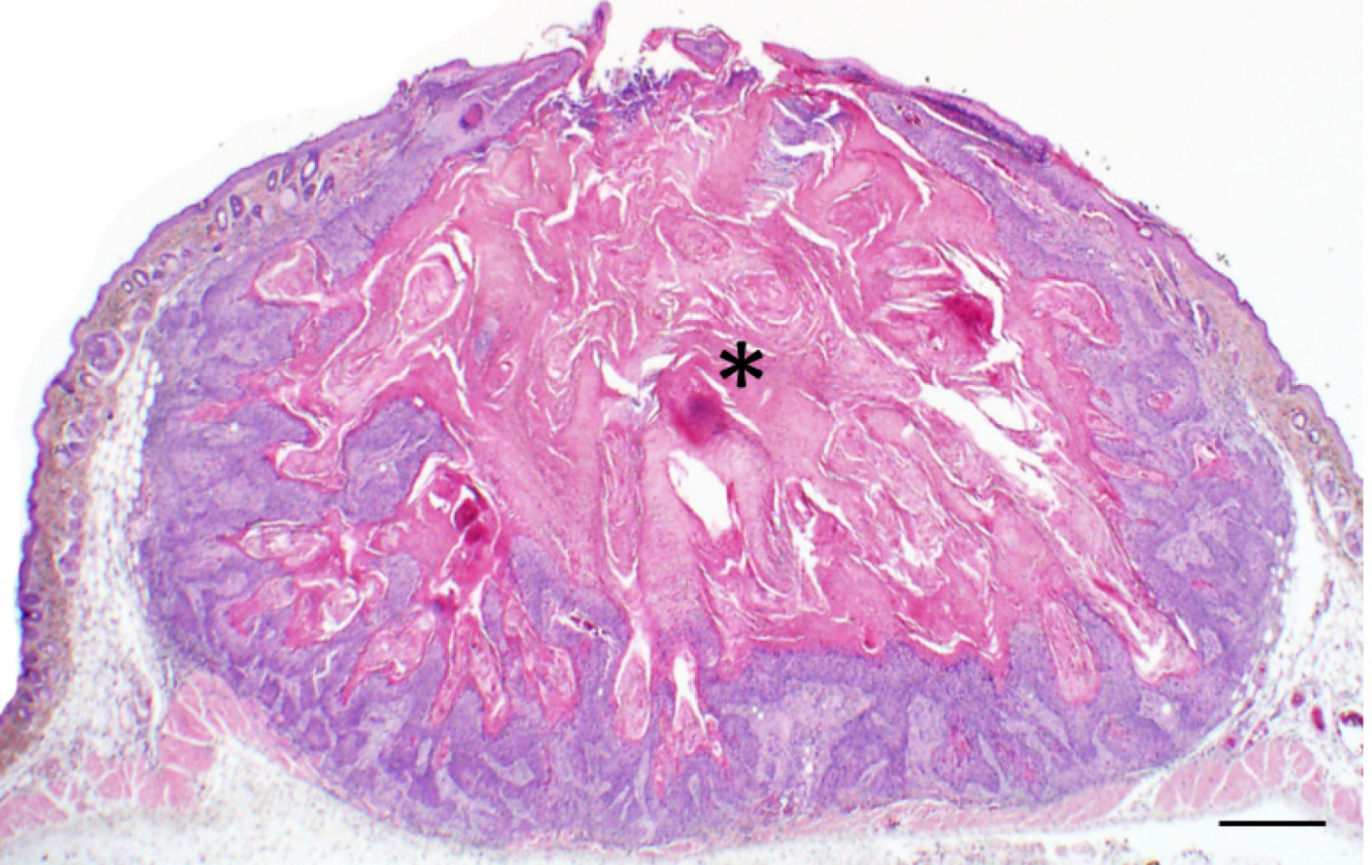
Classic example of a Keratoacanthoma (KA) from a *Zmiz1* mouse. Low magnification photomicrograph showing the crateriform appearance of the KA filled with keratin (asterisk) and bordered by proliferative epithelium. Bar = 500 μm.

Tumors had been collected from Zimz1 mice that ranged in age from 2–12 months (mean 3.6 months); while the range of ages of Rag-SB mice was 6.5–15.5 months (mean 11.6 months). Grossly, KAs from both mouse models progressively developed as focal raised skin tumors. While KAs were generally found on any skin surface, there was a predilection for the back, head, and limbs in *Zimz1* mice and the back and chest in Rag-SB mice (Table 1). These raised nodular lesions appeared as tan to white keratin-filled craters surrounded by raised and moderately erythematous skin margins (Figure 1); very similar to the gross appearance of human KAs [4]. Classical histological descriptions of KAs are characterized by a distinct central crater filled with abundant keratin material (Figure 2). We were then able to histologically classify the KAs into three distinct growth phases.

### 3.2. Growth Phase

**Figure 3:**
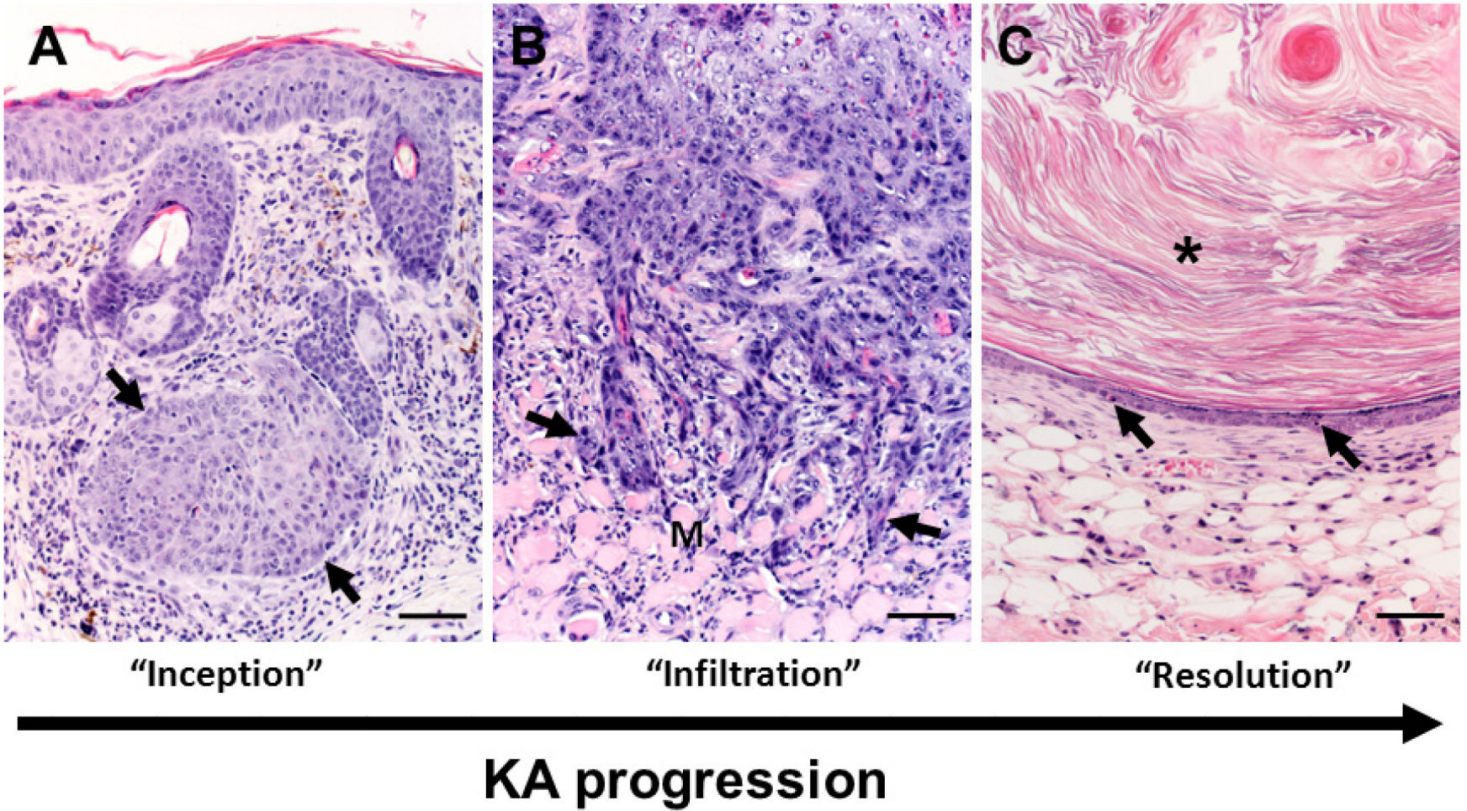
Overview of keratoacanthoma (KA) biology. (**A**) The inception of KA tumors (arrows) occurs in the dermis arising near (in Figure 3A serial sections were not available to confirm continuity with follicle) or from the follicular epithelium. Note: in this tissue section the epidermis is acanthotic (thickened) immediately overlying the tumor with mild dermal inflammation. (**B**) During the “growth” (extending into the “maturation”) phases of KA development, the deep proliferating squamous epithelium can form cellular cords or tongues (arrows) that invade adjacent deeper structures including adipose or skeletal muscle (M) tissues in the panniculus. This example of tumor invasiveness can lead to misdiagnosis of malignant biology. (**C**) In the regression phase, the KA lining epithelium becomes progressively thinner with apoptotic bodies (arrows). As the KA crateriform structure is obscured in regression, the tumor “flattens” into epidermal like structure with smaller mass-like structures often filled by lamellar keratin (asterisk) that resemble benign follicular cysts. Bars = 100 μm.

**Figure 4:**
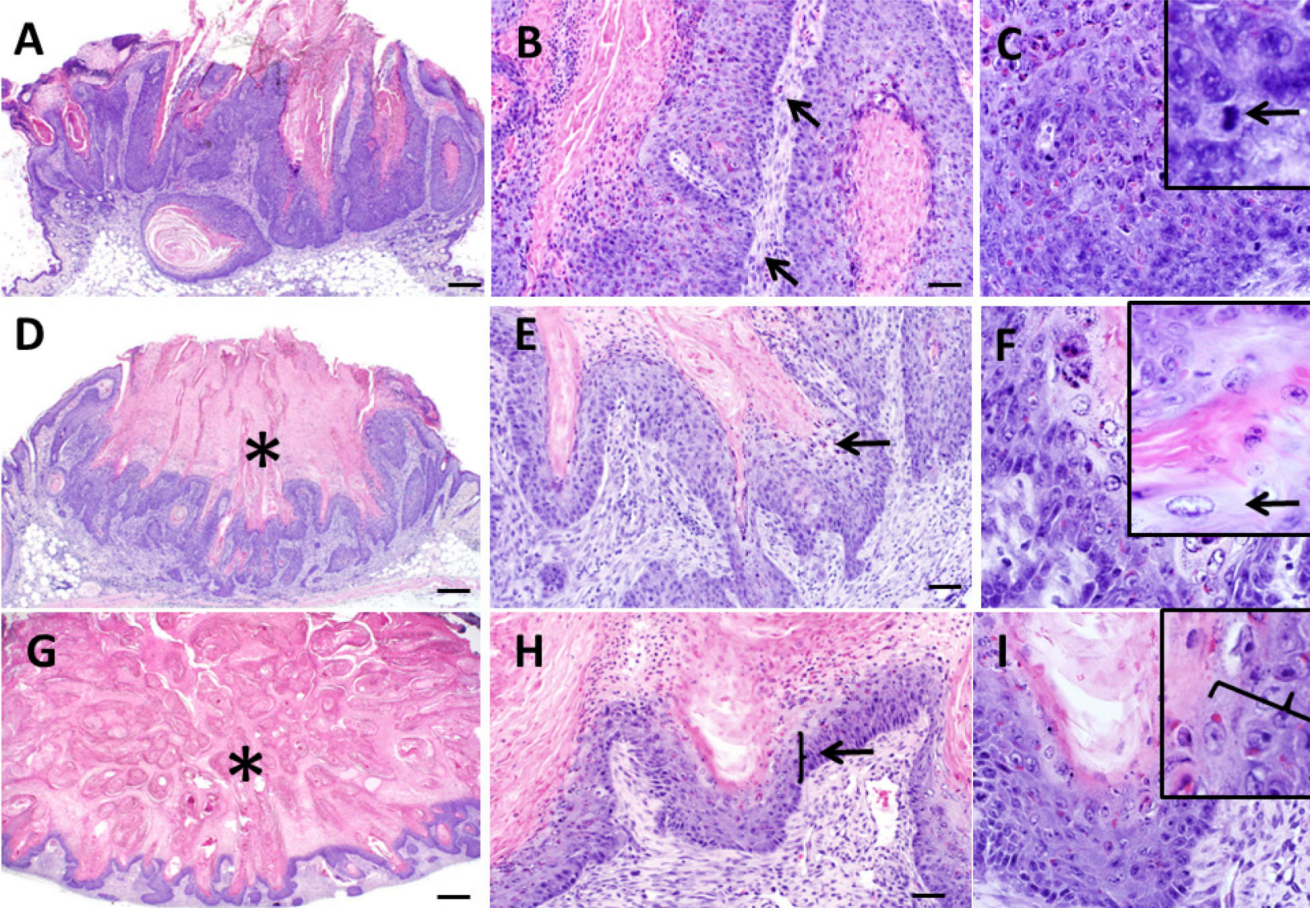
Histopathologic features of keratoacanthomas from *Zimz1* mice. Keratoacanthomas (KAs) were classified into three separate groups based on their morphology on HE section. Those in the “growth” phase (G) (**A**,**B**,**C**) had keratin and inflammation (B, arrows), and were lined by a proliferative layer of keratinocytes which were poorly defined into distinct epidermal layers with anisocytosis and anisokaryosis along with multiple mitotic figures (C, arrows). Those in the “maturation” phase (M) (**D**,**E**,**F**) had a central crater of keratin (D, asterisk) and were lined by proliferative epithelium with patchy regions of “glassy cells” (E, F, arrows) and also had moderate dermal inflammation. Those in the regression phase (R) (**G**,**H**,**I**) had abundant keratin (G, asterisks) with noticeable thinning of the epithelial lining (H, I, arrow) and moderate dermal inflammation. A, D, G bars = 500 μm, B, E, F bars = 50 μm, C, F, I bars = 20 μm.

The inception of KAs typically occurred as a subepidermal solid to crateriform mass originating from or near the follicular epithelium (Figure 3A) which shortly thereafter involves the surface epithelium to create an exophytic mass. The growth phase was characterized by tumors with a thickened wall of acanthotic keratinocytes, nominal to moderate orthokeratosis (Figure 4A) and mixed inflammation (Figure 4B, Table 2). The tumor-associated inflammation in Zimz1 and Rag^+/−^SB mice was composed primarily of scattered to patchy aggregates of lymphocytes and macrophages with fewer neutrophils, eosinophils and rare plasma cells. The RAG^−/−^SB mice lacked T and B cells (data not shown) which is expected as these mice lack mature lymphocytes [13]. The lining keratinocytes had patchy to coalescing fields of pleomorphism with anisocytosis, anisokaryosis and multiple mitoses per high powered field (Figure 4C), which has also been described in human KAs [1]. The deep portion of the mass had cords and trabeculae of neoplastic cells that sometimes showed minor invasion the adipose and skeletal muscle tissues of the panniculus (Figure 3B), but deep invasion and metastasis was not observed.

### 3.3. Maturation Phase

KAs in the mature phase had a distinct central crater that was packed with abundant keratin material (Figure 4D, Table 2). This central crater was lined by an irregular rim of thickened epithelium with multifocal regions of “glassy cells” which are squamous cells with a large amount of very pale eosinophilic cytoplasm and are considered a key diagnostic feature in human KAs [4] (Figure 4E,F). In these murine KAs, glassy cells were less prominent than as those described for human KAs at this stage [4,14]. Inflammation was generally similar to that seen in the growth phase. Mild to moderate dermal fibrosis was noted in some of the tumors in the mature phase. Perineural involvement, which has been documented to occur in human KAs at this stage [4] was not noted in any of the *Zimz1* or Rag-SB KAs examined.

### 3.4. Regression Phase

In the regression phase of development KAs became less crateriform with the lining epithelium becoming increasingly thin (Figure 4G,H, Table 2). This thin epithelium resembled mild to moderately acanthotic epithelium and lacked glassy cells, pleomorphism, and mitotic figures seen in previous phases (Figure 4H,I). Portions of the resolving KA sometimes had a very thin epithelial (epidermis-like) lining surrounding lamellar keratin, morphologically resembling a follicular cyst (Figure 3C). Additional findings in these regressing KAs include mild to moderate fibrosis and in a few cases a moderate mixed inflammatory cell infiltrate that was more prominent than seen in other tumors. Clinically we were not able to study complete regression with return to normal skin morphology because of defined project endpoints requiring euthanasia. It is important to note, evidence of metastatic disease was absent in both models.

### 3.5. Histologic Classification of Murine Keratoacanthomas

**Figure 5:**
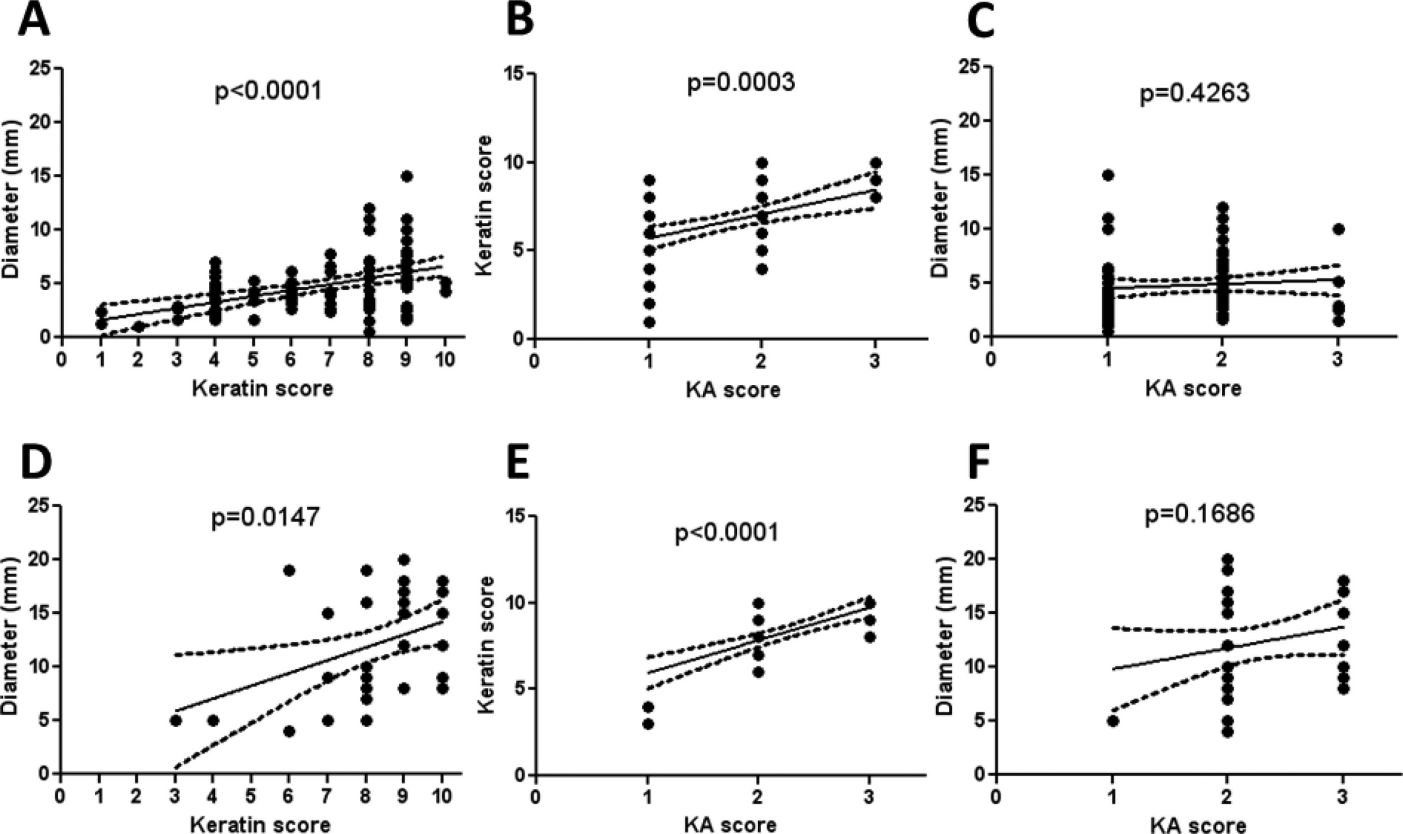
Morphometry and histopathological scoring of KAs. KA diameter was measured and a keratin score was generated by visually determining the amount of keratin present within the KA crater using a score from 0-10. KA score (see Table 2) was also determined. KA diameter and keratin score had a linear correlation in the *Zimz1* KAs (**A**) and RAG-SB KAs (**D**), as did the keratin score and KA score ((**B**,**E**), respectively). There was no correlation in KA score to KA diameter in both the *Zimz1* (**C**) and RAG-SB (**F**).

We observed during the examination of KAs in this study that the largest KAs appeared to have the most abundant keratinization. Because of this, the tumors were scored for keratinization as a percentage of total tumor cross-sectional area, thus designated the “keratin score”. There was a linear correlation between the diameter of both Zimz1 and Rag-SB KAs to the keratin score indicating this initial observation was true (Figure 5A,D respectively). Similarly, there was a linear correlation between the keratin score and the KA score in both models (Figure 5B,E respectively). The KA score was not proportional to the overall diameter of the KA, which was not surprising as resolving KAs were often smaller than those in the maturation phase (Figure 5C,F respectively).

### 3.6. Utility of Immunohistochemistry to Assess Immune Cell Infiltration of Regressing Murine Keratoacanthomas

**Figure 6:**
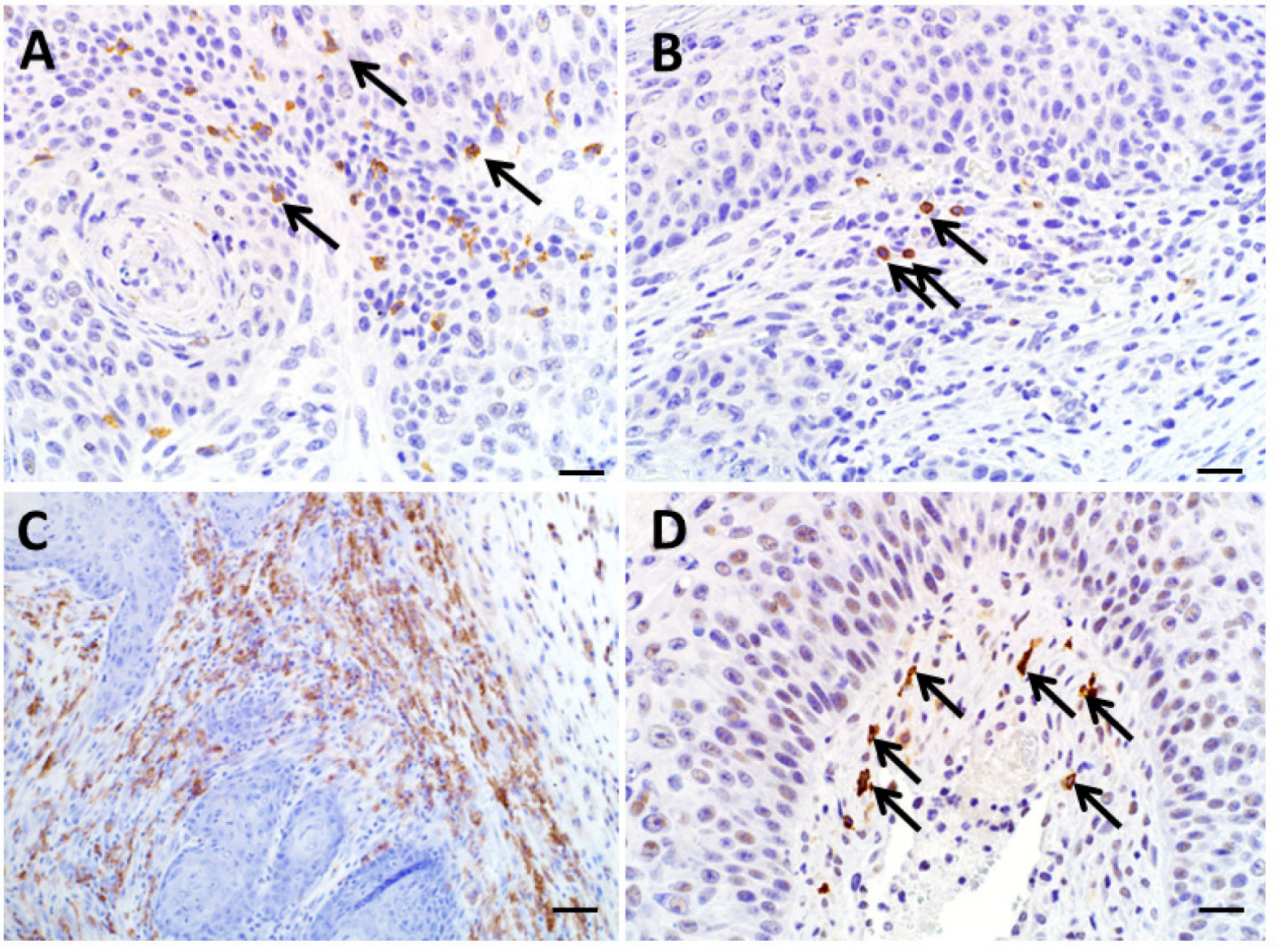
Immunohistochemistry of regressing KAs. (**A**) CD3 immunoreactive cells were noted within the dermis and tumor keratinocyte layer (arrows). (**B**) B220 immunoreactivity was sparse (arrows). (**C**) F4/80 immunoreactivity was common throughout the subepithelial connective tissue of regressing KAs. (**D**) Granzyme B immunoreactive cells were scattered throughout the tumor stroma tissue (arrows). A, B, D bars = 20 μm. C, bar = 50 μm.

We evaluated the utility of detecting immune cell infiltration from 3 KAs in the regression phase. CD3 and fewer B220 immunoreactive cells were found at the peripheral edge of tumors (Figure 6A,B respectively). As mentioned previously, there was an absence of B220 and CD3 immunoreactive lymphocytes in the KAs from Rag2^−/−^ SB mice (data not shown). Within the dermis in regressing tumors from both mouse models there were numerous F4/80 immunoreactive macrophages, which were the overwhelming cell type in most tumors (Figure 6C). Arginase-I immunostaining of macrophages, consistent with M2 phenotype, was scarce in all samples (data not shown). There were moderate numbers of Granzyme B immunoreactive cells, which includes CD8^+^ T cells and natural killer cells, within the dermis and were found primarily at the dermal/epidermal junction (Figure 6D), and also were present interdigitating between keratinocytes. Diffusely the keratinocytes themselves were weakly immunoreactive for NOS2 as has been described [15], but immune cells within the dermis were generally unstained for NOS2 (data not shown).

## 4. Discussion

In the current study, we were able to classify murine KAs into three distinct developmental phases similar to those described for human KAs and experimentally induced KAs in a rabbit model [16]. In humans, KAs are thought to originate from follicular epithelium [17] and this was consistent with our findings in the current study of mouse models. We were also able to study KA regression and immune cell infiltration in select cases and found the immune cell population to be similar to that described for human KAs [13,18]. While these points suggest that the biology of these murine KAs is comparable to humans, there were differences too. The prominent glassy cell layer described as characteristic in human KAs was present in mice and generally restricted to the maturation phase. These glassy cells were less prominent than described for humans [4], which could make diagnosis of murine KAs more challenging for those familiar with human KA morphology. While we cannot say that all murine models with KAs will mimic our findings, this study offers important considerations for future diagnostic efforts studying mouse skin cancer models. Importantly, we show murine KAs follow a developmental pattern similar to that described in human [4] and transgenic rabbit KAs [19] including a regression phase and the aggressive appearance noted in the early development phases did not result in malignancy. Our findings highlight the importance of understanding tumor biology in a new cancer model to ensure that the morphologic diagnosis is consistent and accurate.

We wanted to assess the utility of detecting immune cell infiltration, specifically in regressing KAs. We were able to detect and localize within the tumors a variety of immune cells, suggesting immunohistochemical assessment would be potentially useful in clarifying the role(s) of immunologic-based regression in KAs. During routine assessment of HE stained section, we identified inflammation in all phases of KA development. Therefore, future studies evaluating immune cell infiltration associated with KA regression will need more rigorous approaches. For instance, each developmental phase should be compared for differences in immune cell infiltrates and/or immunohistochemical techniques should be complemented by more sensitive techniques such as flow cytometry to assess subtle shifts in immune cell populations. Interestingly, the immune cell infiltrates were generally seen at each phase of development (including an immunodeficient model) which raises questions about the role of the immune system in regulation of KA regression. Further, another hypothesis for KA regression which does not involve the immune system has been proposed for these follicular tumors. The idea is that growth and regression of these tumors may be under the regulation of factors that govern the normal hair cycle [20]. Future studies in the Zmiz1 model may be able useful to better discern the roles of immune cell infiltration and hair cycle factors in the regulation of regression in these tumors.

In the human literature, there is controversy as to whether KAs are benign squamous proliferations that regress, or if they have potential to become malignant SCC [4,14]. Unfortunately, the distinction between KA and SCC using basic histopathology is not always obvious, and there is a lack of immunohistochemical stains or biomarkers that will distinguish the two tumor types [7]. There also do not appear to be any specific genetic aberrations that aid in diagnosis [21]. As such, KA are often treated clinically as low grade malignancies or potential SCC [14]. In the mouse models utilized in this current study, we did not see progression to SCC or malignant tumors arising from squamous proliferation [22], instead we observed KAs maturing through the three phases of development including regression. In Rag-SB mice we specifically studied KAs, although two skin tumors not included in this study were classified as SCC [9]. This certainly does not imply that KAs progress to SCC as these Rag-SB mice also developed many other types of malignancies [9] and there was not any evidence that these dermal SCC originated from KAs. Importantly, mouse KAs in the current study often had an aggressive appearance (areas of cellular atypia, proliferation and minor invasion) that could lead some observers to presumptively interpret it as SCC. Future studies may include other techniques (e.g., tumor transplantation studies, clonality, *etc*.) to further investigate the pathobiology of these murine KAs.

One note of importance is that these mice were required by animal care guidelines to be sacrificed early because of tumor “burden”. Therefore exhaustive biologic assessment of the whole of the tumor set could not be completed. Accordingly, we cannot completely rule out eventual progression to SCC in some tumors; however, the large portion of KAs that had entered the regression phase did not show evidence for malignant transformation. Given that a large percentage of the KA tumor mass is keratin (similar substance to hair) and that they lack an ulcerated surface, it might be of value to discuss possibility of exemptions to traditional endpoints with Institutional Animal Care and Use Committees. This could allow investigators to further understand the biology of these tumors and result in better defined tumor regression events. These models could offer useful approaches to understanding why KAs develop, why they can appear aggregative yet eventually regress and open the door for new medical/surgical therapies.

## 5. Conclusions

Our study of murine KAs has relevant implications for future investigations. First, the parallel development and biology of KAs of mice and humans suggests the lifecycle of these tumors are regulated by similar factors. Second, translational studies of KAs in mouse models may be useful to investigate mechanisms of tumor regression, novel therapies and potential for malignant transformation. Lastly, the local aggressiveness of murine KAs (which may be misdiagnosed as squamous cell malignancies) highlight the need in experimental models to validate tumor morphology with biology.

## Figures and Tables

**Table 1:** Distribution of keratoacanthomas (KA) tumors on *Zimz1* and RAG-SB mice expressed as a percentage of the total and, in parentheses, the total number identified.

Mouse	Neck	Head	Ear	Chest	Back	Abdomen	Side	Limbs/paws	Tail	Perineal	Total
Zimzl	1% (1)	20% (18)	11% (10)	8% (7)	28% (25)	2% (2)	2% (2)	21% (19)	5% (5)	2% (2)	90
RAG-SB	8% (4)	8% (4)	2% (1)	30% (14)	23% (11)	8% (4)	10% (5)	8% (4)	0% (0)	0% (0)	47

**Table 2:** Phases of murine KA development.

KA Phase	Morphologic features	Transition to next phase
Growth	• Proliferative epidermis with • Anisokaryosis • Anisocytosis • Numerous mitotic figures • Monomorphic/homogenous atypical cells that do not tend to differentiate into normal epidermal layers • Multifocal local invasion of panniculus adipose/muscle • The epithelium commonly forms tongues/cords with scant to variable keratin that is more lamellar and orthokeratotic	• Begins a subtile transition to differentiation of the epidermis with rare keratohyaline granules
Maturation	• Similar to Growth phase with • “Glassy” keratinocytes • Evidence of parakeratotic keratin • Ghost cells • Keratohyalin granules • All consistent with increased differentiation into epidermal layers. • The thickness of the epithelial tongues/cords becomes thinner than in growth phase. • Rarely multifocal local invasion of panniculus adipose/muscle	• Slight overall thinning of the epidermis compared to others within this phase • Slight flattening of the epidermal tongues/cords compared to others within this phase • Fewer “glassy” keratinocytes to be found
Regression	• Loss of tongues/cords/trabeculae • Progressive thinning of the epithelium • The keratin core becomes hollowed out and filled with keratin • Oftentimes acanthotic epidermis • Fibrosis of the subjacent dermis	

**Table 3:** Primary antibodies and their commercially available sources, catalog numbers, dilutions and specific antigen retrieval conditions utilized in the study.

Marker	Antibody	Dilution	Source	Conditions
CD3	Cat# RM-9107-5	1:200	Neomarkers	HIER, citrate buffer (pH 6.0)
B220	Cat# MCA1258G	1:6000	Serotec	HIER, citrate buffer (pH 6.0)
F4/80	Cat# MCAP497	1:6400	Serotec	HIER, citrate buffer (pH 6.0) [11]
Arginase-1	Cat# sc20150	1:300	Santa Cruz	HIER, citrate buffer (pH 6.0) [12]
Nos2	Cat# sc649	1:200	Santa Cruz	HIER, citrate buffer (pH 6.0)
Granzyme B	Cat# ab4059	1:200	Abcam	HIER, citrate buffer (pH 6.0)
